# Supplementary material for: The Neuroprotective Role of Coenzyme Q10 Against Lead Acetate-Induced Neurotoxicity Is Mediated by Antioxidant, Anti-Inflammatory and Anti-Apoptotic Activities
Source: Int J Environ Res Public Health. 2019 Aug 13;16(16):2895. doi: 10.3390/ijerph16162895 (PMC6720293; doi:10.3390/ijerph16162895)

Supplementary Materials

Table S1. PCR effeciency (%).

Actb

Tube	Ct 1	Ct 2	Avg Ct	Sample Quantity	Log (sample quantity)
A	16.31	16.41	16.36	1.0000	0.00
B	19.92	19.79	19.855	0.1000	-1.00
C	23.19	23.12	23.155	0.0100	-2.00

**Dilution Factor**	10

**Slope**	-3.3975
**R Squared**	0.9997
**Efficiency (%)**	**96.94**

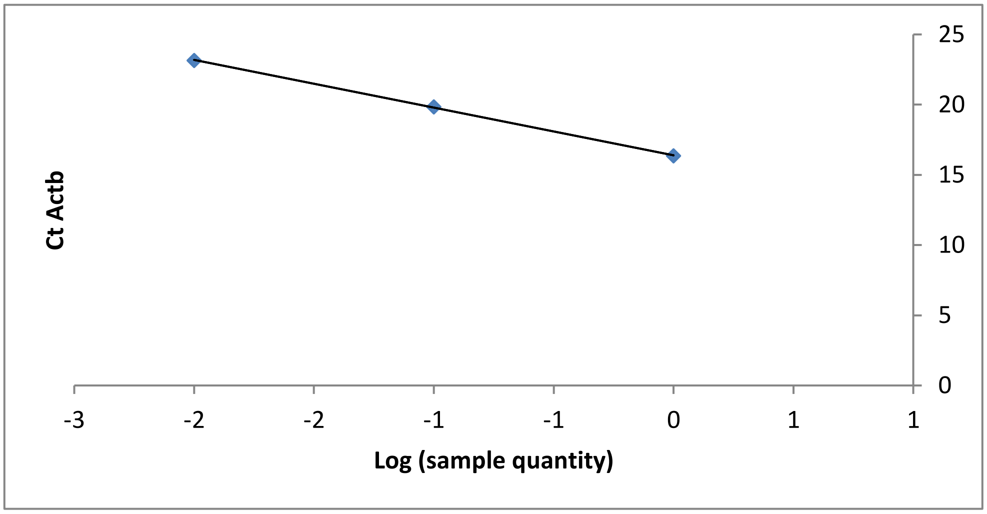

Sod2

Tube	Ct 1	Ct 2	Avg Ct	Sample Quantity	Log (sample quantity)
A	16.51	16.44	16.475	1.0000	0.00
B	19.71	19.59	19.65	0.1000	-1.00
C	22.89	22.74	22.815	0.0100	-2.00

**Dilution Factor**	10

**Slope**	-3.17
**R Squared**	1.0000
**Efficiency (%)**	**106.76**

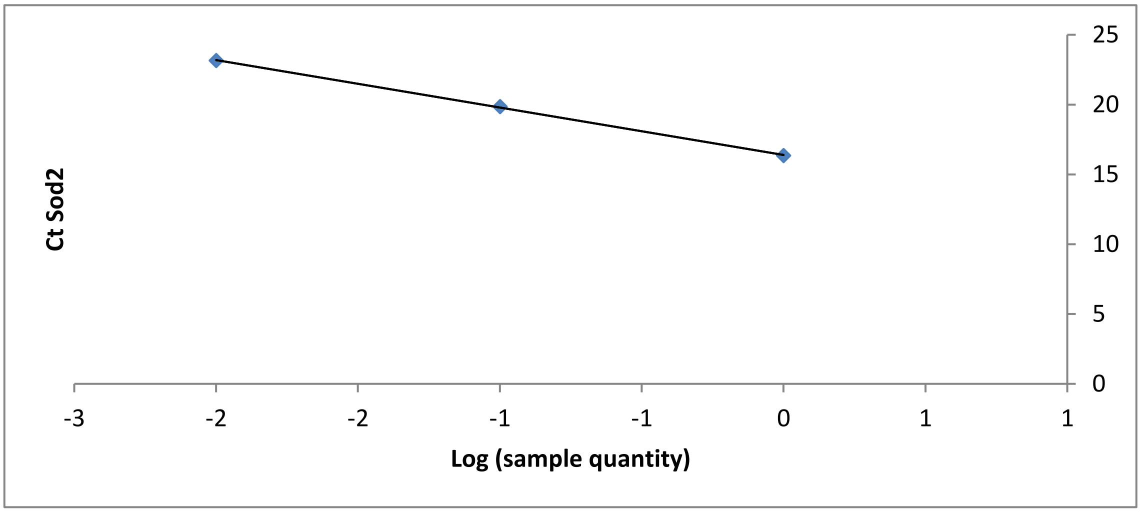

Cat

Tube	Ct 1	Ct 2	Avg Ct	Sample Quantity	Log (sample quantity)
A	20.26	20.14	20.2	1.0000	0.00
B	22.98	23.13	23.055	0.1000	-1.00
C	27.27	27.06	27.165	0.0100	-2.00

**Dilution Factor**	10

**Slope**	-3.4825
**R Squared**	0.9893
**Efficiency (%)**	**93.71**

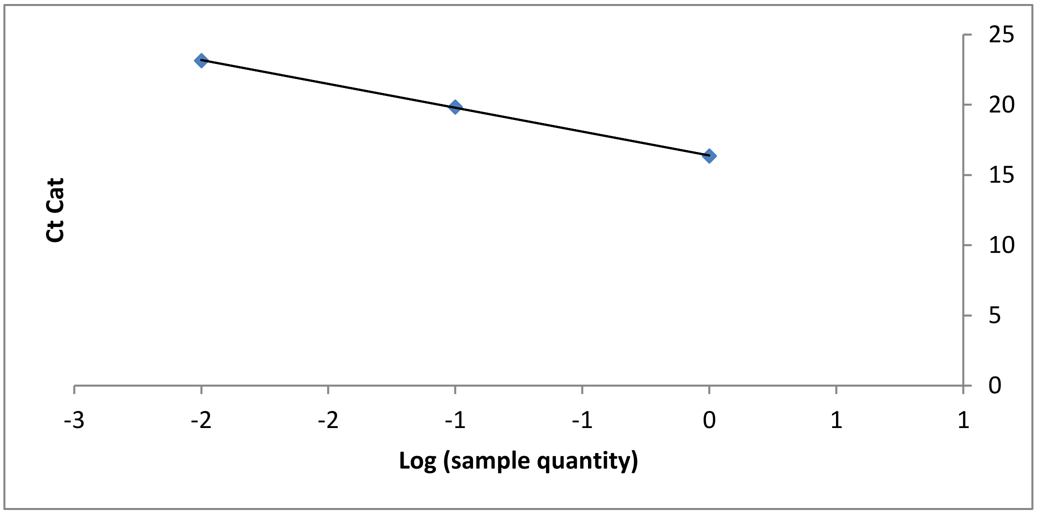

Gpx1

Tube	Ct 1	Ct 2	Avg Ct	Sample Quantity	Log (sample quantity)
A	19.26	19.41	19.335	1.0000	0.00
B	22.27	22.43	22.35	0.1000	-1.00
C	26.04	25.62	25.83	0.0100	-2.00

**Dilution Factor**	10

**Slope**	-3.2475
**R Squared**	0.9983
**Efficiency (%)**	**103.20**

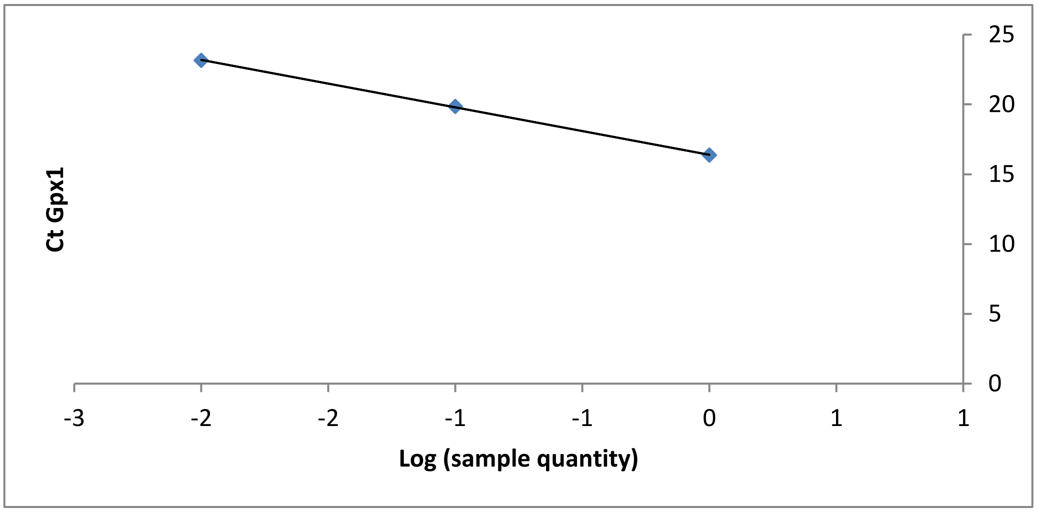

Gsr

Tube	Ct 1	Ct 2	Avg Ct	Sample Quantity	Log (sample quantity)
A	24.61	24.32	24.465	1.0000	0.00
B	26.92	26.41	26.665	0.1000	-1.00
C	30.71	30.18	30.445	0.0100	-2.00

**Dilution Factor**	10

**Slope**	-2.99
**R Squared**	0.9773
**Efficiency (%)**	**116.00**

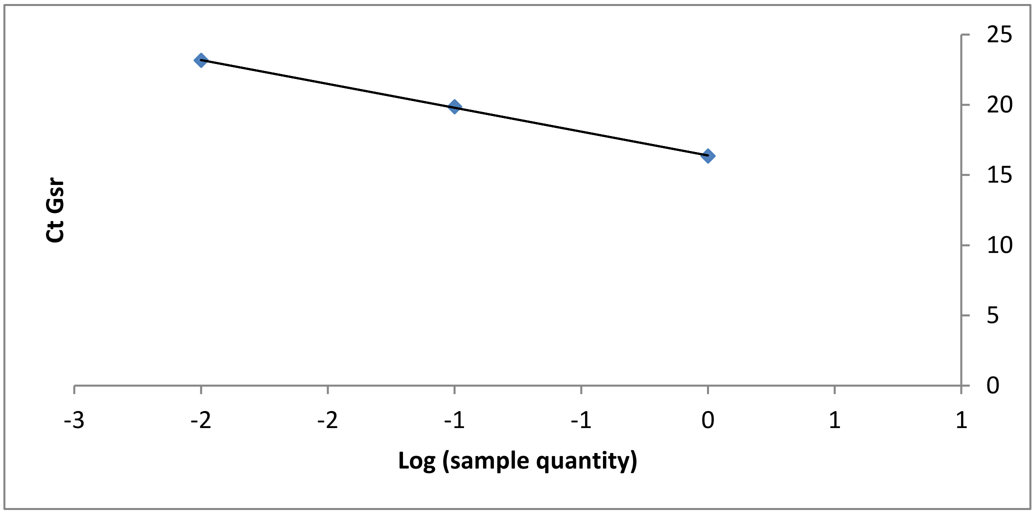

Nrf2

Tube	Ct 1	Ct 2	Avg Ct	Sample Quantity	Log (sample quantity)
A	22.67	22.41	22.54	1.0000	0.00
B	25.18	24.98	25.08	0.1000	-1.00
C	29.06	28.78	28.92	0.0100	-2.00

**Dilution Factor**	10

**Slope**	-3.19
**R Squared**	0.9863
**Efficiency (%)**	**105.82**

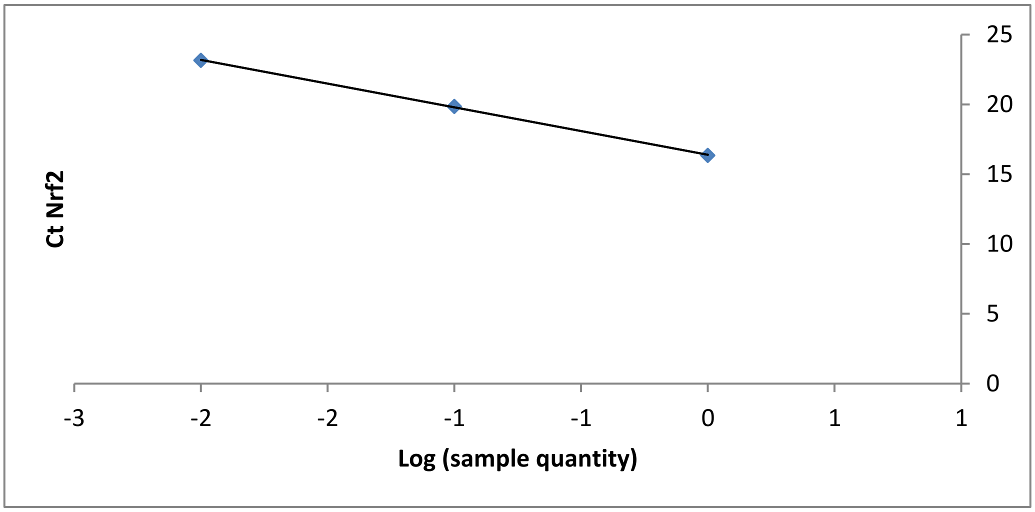

HO1

Tube	Ct 1	Ct 2	Avg Ct	Sample Quantity	Log (sample quantity)
A	19.68	20.03	19.855	1.0000	0.00
B	22.74	22.86	22.8	0.1000	-1.00
C	26.04	26.48	26.26	0.0100	-2.00

**Dilution Factor**	10

**Slope**	-3.2025
**R Squared**	0.9978
**Efficiency (%)**	**105.24**

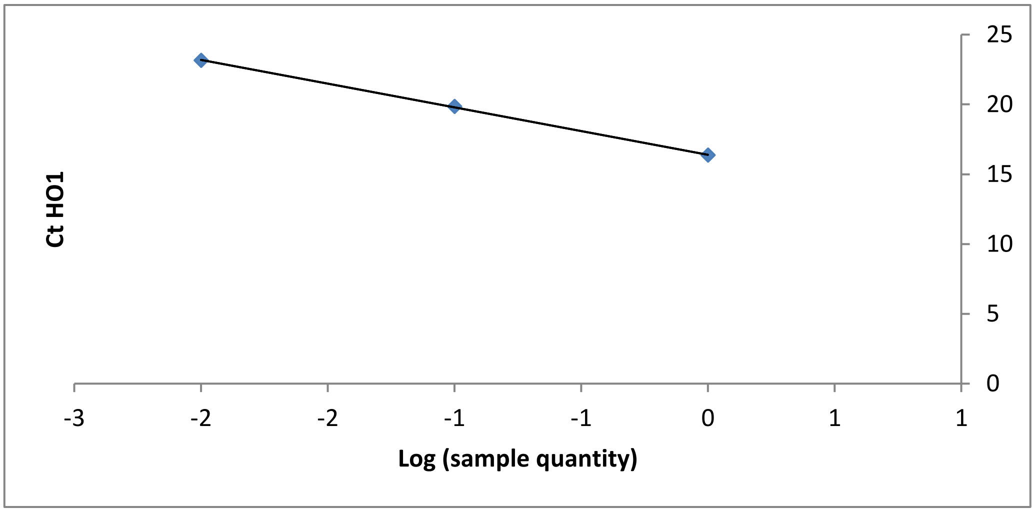

Nos2

Tube	Ct 1	Ct 2	Avg Ct	Sample Quantity	Log (sample quantity)
A	16.78	17.04	16.91	1.0000	0.00
B	20.06	20.31	20.185	0.1000	-1.00
C	23.47	23.84	23.655	0.0100	-2.00

**Dilution Factor**	10

**Slope**	-3.3725
**R Squared**	0.9997
**Efficiency (%)**	**97.93**

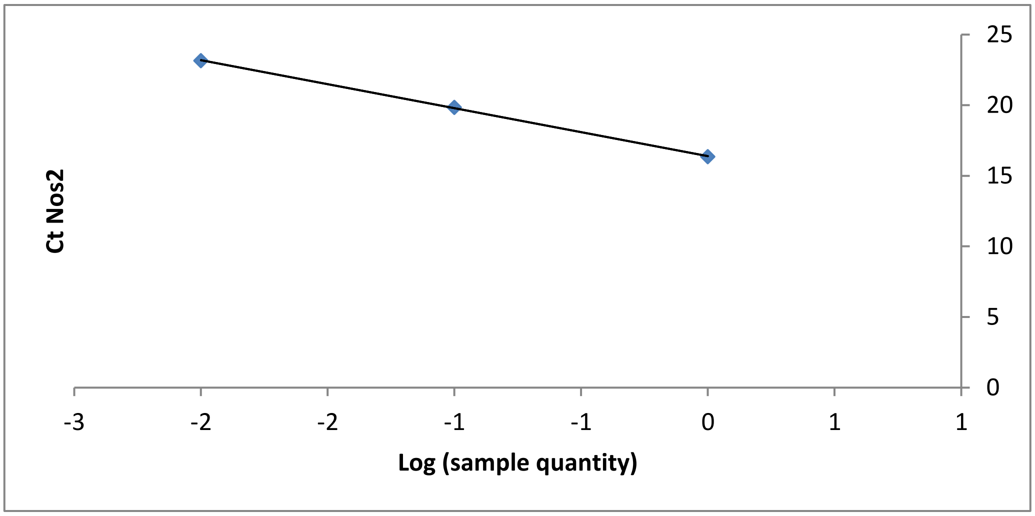

Tnf

Tube	Ct 1	Ct 2	Avg Ct	Sample Quantity	Log (sample quantity)
A	19.87	19.51	19.69	1.0000	0.00
B	22.36	22.42	22.39	0.1000	-1.00
C	26.04	26.2	26.12	0.0100	-2.00

**Dilution Factor**	10

**Slope**	-3.215
**R Squared**	0.9915
**Efficiency (%)**	**104.66**

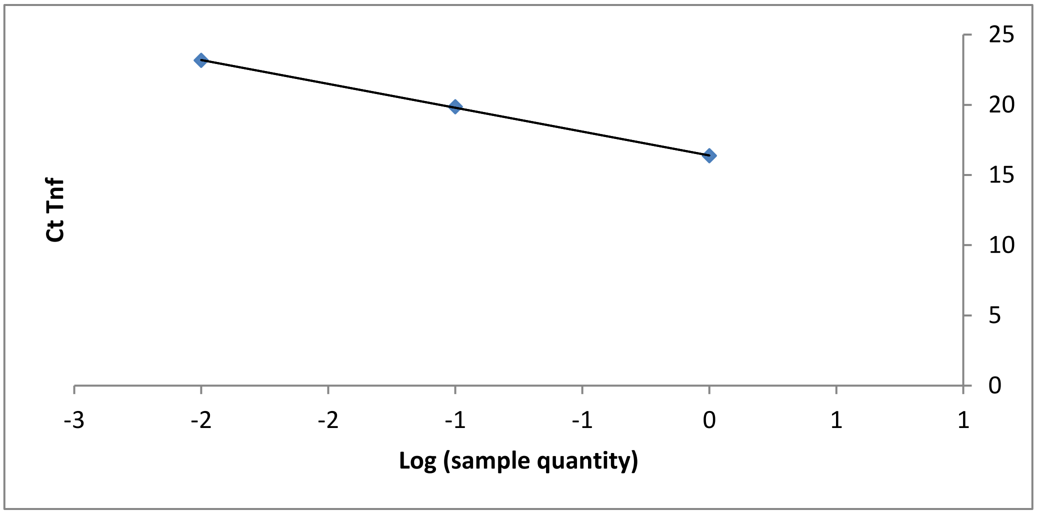

IL1

Tube	Ct 1	Ct 2	Avg Ct	Sample Quantity	Log (sample quantity)
A	17.63	17.53	17.58	1.0000	0.00
B	20.47	20.31	20.39	0.1000	-1.00
C	23.82	23.81	23.815	0.0100	-2.00

Dilution Factor 10

**Slope**	-3.1175
**R Squared**	0.9968
**Efficiency (%)**	**109.30**

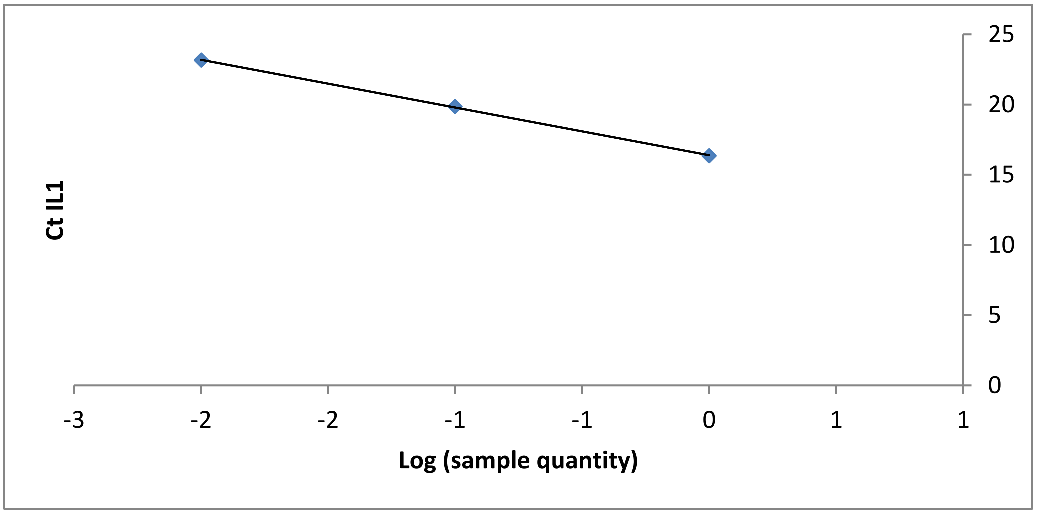

IL10

Tube	Ct 1	Ct 2	Avg Ct	Sample Quantity	Log (sample quantity)
A	18.24	18.12	18.18	1.0000	0.00
B	21.36	21.22	21.29	0.1000	-1.00
C	24.48	24.56	24.52	0.0100	-2.00

**Dilution Factor**	10

**Slope**	-3.17
**R Squared**	0.9999
**Efficiency (%)**	**106.76**

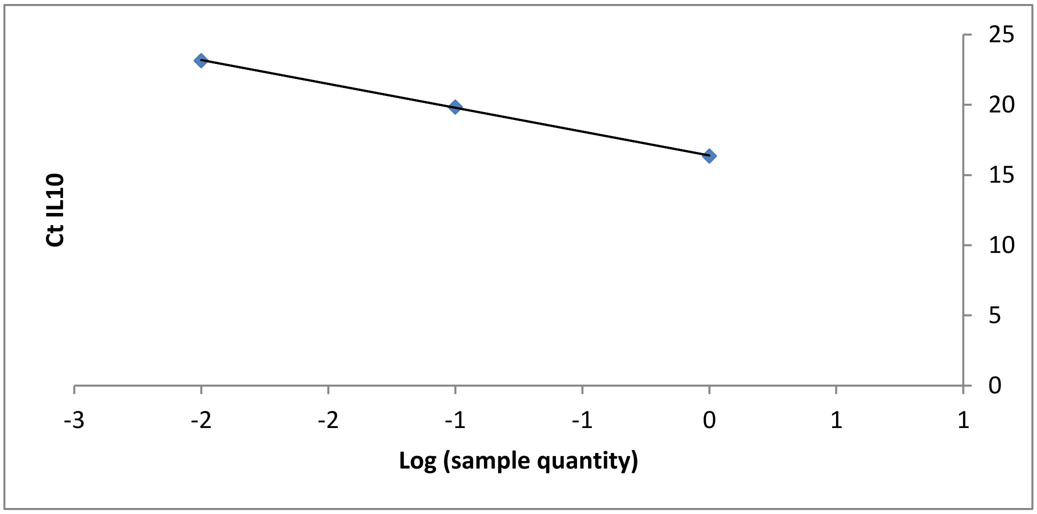

Bcl2

Tube	Ct 1	Ct 2	Avg Ct	Sample Quantity	Log (sample quantity)
A	16.98	17.03	17.005	1.0000	0.00
B	20.36	20.24	20.3	0.1000	-1.00
C	23.84	23.79	23.815	0.0100	-2.00

**Dilution Factor**	10

**Slope**	-3.405
**R Squared**	0.9997
**Efficiency (%)**	**96.65**

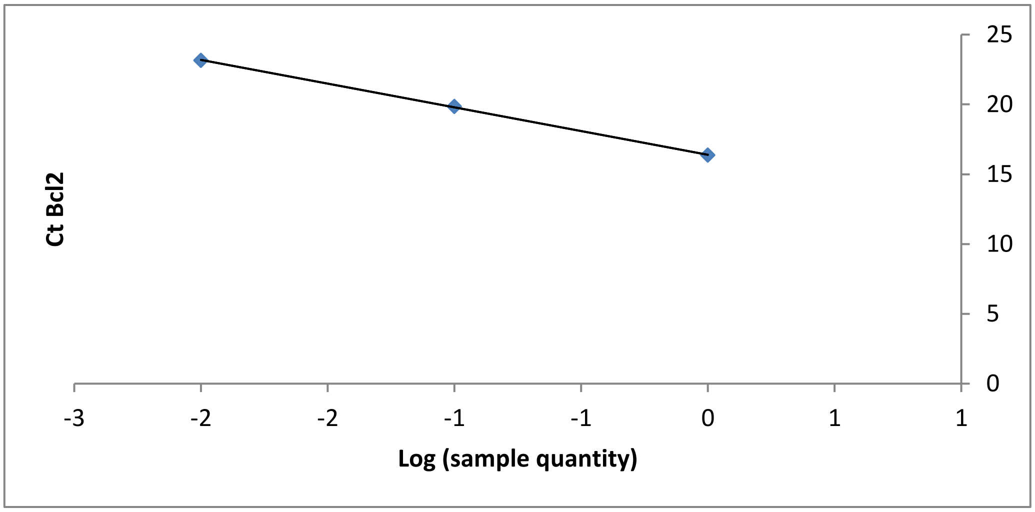

Bax

Tube	Ct 1	Ct 2	Avg Ct	Sample Quantity	Log (sample quantity)
A	17.42	17.49	17.455	1.0000	0.00
B	20.36	20.24	20.3	0.1000	-1.00
C	23.89	24.06	23.975	0.0100	-2.00

**Dilution Factor**	10

**Slope**	-3.26
**R Squared**	0.9946
**Efficiency (%)**	**102.65**

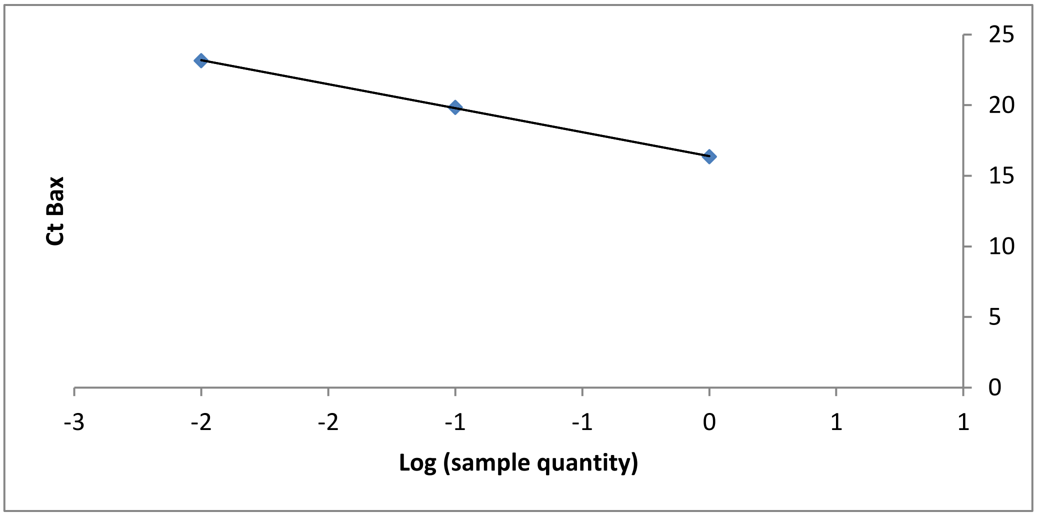

Casp3

Tube	Ct 1	Ct 2	Avg Ct	Sample Quantity	Log (sample quantity)
A	21.69	21.74	21.715	1.0000	0.00
B	24.68	24.71	24.695	0.1000	-1.00
C	27.93	28.04	27.985	0.0100	-2.00

**Dilution Factor**	10

**Slope**	-3.135
**R Squared**	0.9992
**Efficiency (%)**	**108.44**

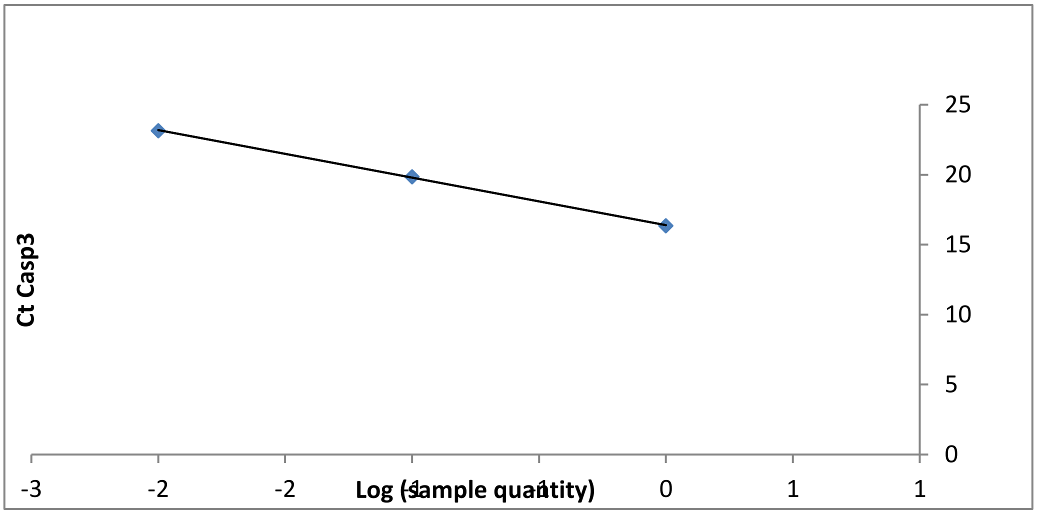

Supplement: Supplementary file 1 [file ijerph-16-02895-s001.pdf]
